# Isolation and Characterization of Sixteen Polymorphic Microsatellite Loci in the Golden Apple Snail *Pomacea canaliculata*

**DOI:** 10.3390/ijms12095993

**Published:** 2011-09-16

**Authors:** Lian Chen, Haigen Xu, Hong Li, Jun Wu, Hui Ding, Yan Liu

**Affiliations:** 1 Nanjing Institute of Environmental Sciences under Ministry of Environmental Protection, 8 Jiangwangmiao Street, Nanjing 210042, Jiangsu, China; E-Mails: chenlian_2004@163.com (L.C.); wujun@nies.org (J.W.); nldinghui@gmail.com (H.D.); liuyan@nies.org (Y.L.); 2 Jiangsu Key Laboratory for Biodiversity and Biotechnology, College of Life Sciences, Nanjing Normal University, 1 Wenyuan Road, Nanjing 210046, Jiangsu, China; E-Mail: hongli82@yahoo.cn

**Keywords:** golden apple snail, invasive species, microsatellite loci, *Pomacea canaliculata*

## Abstract

We report the characterization of 16 polymorphic microsatellite markers in the golden apple snail, *Pomacea canaliculata*, a pest registered in the list of “100 of the world’s worst invasive alien species”. The fast isolation by AFLP (Amplified Fragment Length Polymorphism) of sequences containing repeats (FIASCO) method was used to isolate microsatellite loci, and polymorphism was explored with 29 individuals collected in an invasive region from China. These primers showed a number of alleles per locus ranging from three to 13. The ranges of observed and expected heterozygosity were 0.310–0.966 and 0.523–0.898, respectively. These microsatellite markers described here will be useful for population genetic studies of *P. canaliculata*.

## 1. Introduction

The invasive species *Pomacea canaliculata* (Lamarck 1822) (Caenogastropoda, Ampullariidae), commonly known as the golden apple snail, is native to freshwater wetlands of South America [1], in the area extending from the La Plata river basin southwards to the Tandilia and Ventania mountains (Southern Pampas, Argentina) [2]. *P. canaliculata* is gonochorism. Females are homogametic (XX), while males are heterogametic (XY) [3]. The species has been introduced into several southern and eastern Asian countries since the 1980s [4], becoming a serious pest causing great damage to agricultural plants and macrophytes in non-agricultural wetlands [1,4]. *P. canaliculata* is the only aquatic snail listed as one of the 100 of the world’s worst invasive alien species by the IUCN Species Survival Commission Invasive Species Specialist Group [5]. Since its introduction to China for aquaculture in the 1980s, its rapid spread has caused ecological problems and great economic losses in southern provinces of China [6]. It has been listed as one of the 16 invasive species in China by the State Environmental Protection Administration of China.

However, only a few genetic studies have been conducted to decipher the evolutionary processes associated with the invasion of *P. canaliculata* [4,7]. Most previous studies of *P. canaliculata* in China have focused on its distributions, local pernicious effects and issues related to chemical control. In the case of invasive species, the study of their genetic diversity in the invaded area in comparison to the native area could help to infer important aspects of the invasion process, like the route(s) of invasion, the time of invasion, *etc*. Microsatellite markers are useful tools to investigate the genetic diversity in wild populations because they are highly polymorphic and codominant. However, reports on the development, characterization and use of microsatellite loci in *P. canaliculata* are still scarce. The present study provides 16 polymorphic microsatellites isolated from *P. canaliculata,* which will allow investigating genetic diversity and population genetic structure of *P. canaliculata* in its native and invasive range, as well as tracing its invasion history.

## 2. Results and Discussion

A total of 80 recombinant clones were sequenced. From the 30 successfully amplified primer pairs, 16 loci showed polymorphism in the 29 individuals. The number of alleles per locus ranged from three to 13, whereas the observed and expected heterozygosities ranged from 0.310 to 0.966 and from 0.523 to 0.898, respectively (Table 1). Nine loci (Pc59, Pc83, Pc88, Pc97, Pc102, Pc140, Pc156, Pc221, Pc235) deviated from the Hardy–Weinberg equilibrium in the tested population after Bonferroni correction [8]. Analysis with MICROCHECKER [9] indicated the possible occurrence of null alleles at four of the microsatellites (Pc59, Pc97, Pc102, Pc140). Stuttering was found in two loci (Pc59 and Pc140) confirmed by MICROCHECKER [9]. Null alleles are found in most taxa [10], but seem to be particularly common in populations with high effective population size [11] such as mollusks [12,13]. The presence of null alleles can sometimes be detected as an excess of homozygotes leading to deviations from Hardy–Weinberg expectations. Null alleles create false homozygotes, they are problematic for parentage analysis [14]. In addition, null alleles lower apparent genetic variability, they may erroneously inflate levels of genetic differentiation and affect population genetic analyses that rely on HWE [15,16]. However, there are several statistical approaches used for correcting allele frequencies for null alleles [16,17]. A deviation from HWE may also be due to selection, population mixing, nonrandom mating [18], sampling strategies, and undetected sex-linkage. Redesigning the primers for these loci [12] and further population genetic studies will be helpful to clarify this question. No significant pairwise linkage disequilibrium was shown between any of the loci after Bonferroni correction [8], indicating the independent behavior of all loci.

## 3. Experimental Section

### 3.1. Isolation of Microsatellite Markers

Genomic DNA was extracted from foot tissue of one individual collected from Zhaoqing City of Guangdong Province in China using the DNeasy Tissue Kit (QIAGEN). About 250 ng DNA was digested using M*se* I and ligated to double-stranded M*se*I linker (M*se*I F: 5′-TACTCAGGACTCAT-3′ and M*se*I R: 5′-GACGATGAGTCCTGAG-3′) [19] for 3 h at 37 °C. The digested-ligated product was then amplified using an M*se*I-N primer (5′-GATGAGTCCTGAGTAAN-3′) [20]. After denaturation of 5 min at 95 °C, amplified product was hybridized with 5′-biotinylated (CA)_15_ probe for 1 h at 65 °C. Hybridized probe-DNA was captured using streptavidin-coated magnetic beads (Streptavidin magnesphere Paramagnetic Particles, Promega). Nonspecific binding and unbound DNA were removed by several nonstringent and stringent washes. These microsatellite-enriched DNA fragments were PCR-amplified again and then ligated into pGEM-T Easy vectors (Promega) and transformed into JM109 competent cells (Takara). Recombinant clones were identified using blue/white screening on Luria–Bertani agar plates containing ampicillin, X-gal and IPTG. Insert positive bacterial clones were amplified using one primer for the vector and a second repeat-containing oligonucleotide. Positive clones were sequenced using an ABI PRISM 3730 automated sequencer (Applied Biosystems). Primer sets were designed for microsatellite sequences using PRIMER 3 [21].

Levels of locus polymorphism were assessed in 29 assumed unrelated individuals of *P. canaliculata*, sampled from two sites of Guangdong Province (Guangzhou City and Zhaoqing City), in the southern part of China. PCR amplifications were conducted using the following conditions: an initial denaturation step at 95 °C for 5 min, followed by 30 cycles of 30 s at 95 °C, 30 s at the locus specific optimal annealing temperature (see Table 1), and 30 s at 72 °C, followed by a final extension of 10 min at 72 °C. Each reaction of 15 μL contained 20 ng template DNA, Ex Taq premix buffer 7.5 μL (Takara), and 0.5 pmol of each of forward and reverse primers, forward primers were labeled with 6FAM, HEX or TAMRA. Labeled fragments were discriminated using capillary electrophoresis on an ABI PRISM 3730xl DNA Analyzer (Applied Biosystems), and allele sizes were determined using GENEMAPPER version 4.0 (Applied Biosystems).

### 3.2. Data Analysis

Levels of expected and observed heterozygosities were calculated using CERVUS 3.0 software [22]. Tests for deviations from Hardy–Weinberg equilibrium (HWE) and linkage disequilibrium (LD) at each locus were performed in GENEPOP version 4.0 [23]. Results of tests for linkage and Hardy–Weinberg disequilibria were corrected for multiple comparisons by applying sequential Bonferroni corrections [8]. The MICRO-CHECKER [8] analysis was used to estimate the most probable cause of departures from HWE.

## 4. Conclusions

The microsatellites described here will be useful for investigating the genetic diversity, in particular the genetic structure within populations of *P. canaliculata*, which should help improve management strategies for this species.

## Figures and Tables

**Table 1:** Characteristics of the 16 microsatellite loci isolated from *P. canaliculata*: locus name, primer sequences, repeat motif, allele sizes, annealing temperature (*T*_a_), number of alleles (*N*_a_), observed (*H*_O_) and expected (*H*_E_) heterozygosities, and GenBank Accession no.

Locus	Primer sequence (5′-3′)	Repeat motif	Allele size range (bp)	*T*_a_(°C)	*N*_a_	*H*_O_	*H*_E_	Accession No.
Pc46	F: (HEX)CTGCTCACTCAGCCATTC R: GCTTACCACACCCTTAGA	(CA)_14_	141–165	55	13	0.86	0.90	JN129127
Pc51	F: (6FAM)AGCATCTGTGGGAAAGGTGAC R: GCCAGCAGCAAGTAATGTGAG	(CA)_9_CG(CA)_6_	164–176	55	7	0.55	0.75	JN129128
Pc59	F: (TAMRA)GCGATACTTTACGGACTTG R: CAAAATATGCTTTCATCTGC	(CA)_24_	131–173	55	6	0.31	0.80	JN129129
Pc69	F: (6FAM)TGGTAAAGGGTTTGGGTCGTC R: GGGAATAGGGACAGTTGAGAGG	(CA)_8_AA(CA)_7_	117–129	55	5	0.45	0.52	JN129130
Pc82	F: (6FAM)CAAGCGAGTATTTCAGT R: ACCTCAATGTAATCACG	(CA)_6_CGCCTA(CA)_9_	204–218	50	6	0.69	0.77	JN129131
Pc83	F: (HEX)CACTGTATCATCCCCTG R: TCTGGTTGAGTTTCTACG	(CA)_13_	187–199	50	6	0.83	0.78 *	JN129132
Pc88	F: (HEX)GATGTAAGTGTGCTTTCAAC R: AGGGTTCGGAGACAGAC	(CA)_12_	170–184	55	5	0.90	0.64 *	JN129133
Pc97	F: (HEX)TTCCACAACCATCATCACG R: CTCGGGGTCACACTTCTG	(CA)_13_	129–145	55	6	0.45	0.81 *	JN129134
Pc102	F: (HEX)ACGGCTTCCAACTCAGA R: TGCTTTCCTTTAGTCCAG	(CA)_14_	166–196	55	6	0.41	0.74 *	JN129135
Pc113	F: (6FAM)TGCGTTTACTGGGAGAAG R: GCATAATCGGGGAAGAAG	(CA)_13_CT(CA)_12_	208–256	55	7	0.76	0.81	JN129136
Pc140	F: (6FAM)ACACCTTTTCCGACACG R: GAGACGCTTTGACCACAT	(CA)_6_CT(CA)_5_	195–217	52	6	0.31	0.73 *	JN129137
Pc156	F: (TAMRA)ACCTTGTCCAGTTCTTC R: GAAATAGTCCTAAGTCCTC	(CA)_18_CG(CA)_23_	170–190	52	9	0.97	0.79 *	JN129138
Pc205	F: (6FAM)CCTCTTCAGTGTTTGGAC R: ATACAGCAGGGTGGGAT	(CA)_7_-(CA)_11_	110–128	52	8	0.79	0.76	JN129139
Pc216	F: (6FAM)TGCCCCAGCTTTGTAAA R: CTCTCCTCCCTGCTCTATG	(CA)_5_AGCA AA(CA)_11_AGCAAA(CA)_7_	123–129	52	3	0.45	0.53	JN129140
Pc221	F: (HEX)ATGGCGAACACCAACTC R: TACTTCACGCATGCTTTG	(CA)_29_AA(CA)_8_	172–194	55	10	0.93	0.87 *	JN129141
Pc235	F: (TAMRA)AACCAACTAACAAACTCG R: GCAAAGGTAGTGTCCAT	(CA)_30_	175–199	50	9	0.93	0.73 *	JN129142

* indicates significant deviations from Hardy–Weinberg equilibrium after sequential Bonferroni correction.
